# Is the Bacillus Tuberculosis the Cause of Tuberculosis?

**Published:** 1913-12

**Authors:** 


**A Monthly Review of Medicine and Surgery**

EDITOR AND PUBLISHER DR. A. L. BENEDICT, 228 Summer St. corner of Elmwood Ave., Buffalo. (Address for all communications. Please make personal and telephone calls before 1 P. M.)

Third, in age, in the western continent. Independent but supports professional organization. News limited mainly to western N. Y. and Penn.
Subscription $2.00 a year; $3.00 for two years in advance. Changes and errors in address or failure or duplication of delivery, should be reported immediately.

YEARLY VOLUME 69 DECEMBER, 1913 No. 5

### Is the Bacillus Tuberculosis the Cause of Tuberculosis?

We raise this question not in the bacteriologic sense, but as a live issue in practical prophylaxis—a live issue because the medical press is replete with articles which, without in any way denying the existence, identity and infectivity of the bacillus, more or less explicitly minimize the importance of measures to prevent foci of infection and lay all the stress of prophylaxis and therapeutics upon the raising of resistance.

An old politician was asked if the fact that he was regarded as a dangerous radical at the outset of his career and as an ultra-conservative in his later life, did not indicate a process of gradual maturing and gain in wisdom. He replied that his views had remained fixed but that popular opinion had changed. The same question and answer might apply to the writer with regard to tuberculosis. It is not intended, however, to urge the scientific conception of the bacillus as the essential and practically operative cause of tuberculosis. This task might better be left to those more expert and more directly concerned with tubercular cases. Nor would the writer give the impression of ignoring the importance of means of increasing resistance. Indeed, whatever cases he has undertaken, have been with the full understanding that he did not regard the present so-called antiseptic measures as of much value, that he would not attempt to treat the lungs or other part involved, that he did not regard methods of conferring specific immunity as yet in a practical stage, that he had no personal views as to hygiene different from the prevalent ones, except that he would not expose a sick person to inclement weather liable to be dangerous to those in health; in short, that the treatment should depend mainly upon careful nutrition. By careful nutrition, is meant a properly balanced diet, adapted so far as possible to the needs of the particular patient, and not implying an overdose either of any one organic food stuff or of all.

The following table, prepared by Biggs, shows that there has been a decided diminution in tubercular mortality. It may be questioned whether this reduction is due to what may be called germ prophylaxis or to hygiene. It is evident, on a little reflexion, that the marked decrease in the general death rate, due largely to prevention of traumatisms, typhoid and acute infections generally, individual improvements in therapeutic measures, etc., would, if anything, increase the tubercular mortality, by saving lives to succumb to this disease. On the contrary, Biggs's statistics show that, on the whole, the tubercular mortality has declined more rapidly than the general death rate. This can be due to only two general factors, germ prophylaxis and personal hygiene. Personal hygiene may be facilitated by general sanitary measures, but these measures must be reduced to one or the other of these factors.

It is impossible at present to prove statistically which of these two factors deserves the credit. Habits of exercise and ventilation have improved in recent years. Hours of labor and lighting have been improved. There has been considerable nominal improvement in foods. On the other hand, there has been a relative increase in indoor work, a considerable condensation of population, especially in cities which are the basis of Biggs's statistics, and a very marked increase in the cost of food. Provision for incipient and suspected tuberculosis has increased, but it is doubtful whether to such an extent as to have influenced the statistics given.

With this balance of hygienic factors and the difficulty of drawing conclusions, it is, to say the least, advisable to continue strenuous efforts at germ prophylaxis. In Cleveland, careful study has shown that every death from tuberculosis corresponds to eight living foci of infection. It is very easy to say that, unless we can eradicate every fomes, there is no use in bothering with any; that the predisposed individual will pick up his germ somewhere and sometime. The fact remains that, largely owing to the disinfectant properties of sunlight and the relatively small chances of dissemination of bacilli except in dust or by gross contact with infectious masses, the striking distance of a case of tuberculosis is short. Another important point to remember is that both infectivity and prophylaxis operate in geometric not arithmetic progression. Every case tends to produce not one but several, and each of these several more, and so on, up to the point at which lines of communication cross or the entire susceptible part of the population becomes tuberculous. It is altogether probable that this point was actually reached in our country, when the ordinary minimum general mortality was about 20:1000 and the tubercular mortality about 2:1000. On the other hand, the safeguarding of one case of tuberculosis means the successive escape of several, several times several. . . . . . individuals subject to the diminishing occurrence of crossed lines of communication, until the final limit is reached of a minute incidence among the highly susceptible by the probably inevitable occasional residue of fomites.

We have recently seen several medical articles emphasizing the importance of measles, pertussis and influenza as predisposing to tuberculosis, and we wish to point these articles with a new moral: that a large share of susceptibility to tuberculosis is a transient condition and not inherent and permanent in the individual. If we can, by various systems of quarantine (using the word in an elastic sense) prevent the contact of tuberculous and non-tuberculous individuals for any appreciable number of the latter or for any appreciable period of unusual susceptibility, we can diminish tuberculosis at a rate that will increase until the minimum is reached. If we can diminish, by methods of disinfection, the gross amount of infectious matter so that, by the law of chance, there will be certain places free from it and so that the general environment of the average person will be free from it for appreciable periods—one windy, dusty afternoon, for example—we can eradicate the starting point of other geometric series of infectious transmission.

Remember that we are dealing, save for the interfering factors mentioned, with decreasing geometric series. With the exception of Chicago, where there has been a considerable alteration in conditions, the various cities mentioned show absolute tubercular mortalities varying from a little over 6/10 to a little less than 9/10 of what they were ten years previously. This diminution does not, of course, represent one step in a geometric series, but several steps at very irregular intervals. On the average, it is probably about three years from the death of one case to the death of the cases directly dependent upon it, so that the change for the decade represents approximately three terms in a descending geometric series. However, for our purposes, we may take the decline for a decade as a single term in a larger series. If the same rate of progress were maintained, the statistics for 1921 should show tubercular mortality rates of from 9/10 x 9/10 down to 6/10 x 6/10 of those in 1901. If anything approaching such results happened, it would be an absolutely convincing argument in favor of germ prophylaxis as against personal hygiene, since it is not conceivable that susceptibility could be thus diminished by heredity in the space of a third of a single generation nor practically possible that there could be general improvements in personal hygiene acting thus rapidly in the face of condensing population and higher cost of living.

Is it not worth the trouble and expense of quarantine and disinfectant measures (using these terms broadly) to determine whether this theory is correct or not?

| City | 1901 General Death Rate | 1901 Ratio | 1901 Tuberc. Death Rate | 1911 General Death Rate | 1911 Ratio | 1911 Tuberc. Death Rate |
|---|---|---|---|---|---|---|
| Paris ...... | 18.2 | 4.2:1 | 4.32 | 16.7 | 4.6:1 | 3.59 |
| New York.. | 19.91 | 8.7:1 | 2.29 | 15.13 | 8.5:1 | 1.76 |
| Boston ..... | 19.87 | 8.4:1 | 2.36 | 17.08 | 11:1 | 1.55 |
| Philadelphia. | 18.26 | 8.2:1 | 2.23 | 16.50 | 8.8:1 | 1.87 |
| Chicago .... | 13.93 | 9.8:1 | 1.42 | 14.55 | 8.7:1 | 1.66 |
| London .... | 17.24 | 9.5:1 | 1.81 | 12.71 | 11:1 | 1.14 |
| Berlin ..... | 18.00 | 8.2:1 | 2.18 | 14.65 | 8.2:1 | 1.78 |

